# Novel targeted treatments in hairy cell leukemia and other hairy cell-like disorders

**DOI:** 10.3389/fonc.2022.1068981

**Published:** 2022-12-22

**Authors:** Elsa Maitre, Jerome Paillassa, Xavier Troussard

**Affiliations:** ^1^ Hématologie, Centre Hospitalier Universitaire Caen Normandie, Avenue Côte de Nacre, Caen, France; ^2^ Service des Maladies du Sang, Centre Hospitalier Universitaire d’Angers, Angers, France

**Keywords:** Hairy cell leukemia, HCL, splenic B-cell leukemia/lymphoma with prominent nucleoli, BRAF inhibitors, BTK inhibitors, anti-CD20 monoclonal antibodies, *Moxetumomab pasudotox*, new drugs

## Abstract

In the category of mature B-cell neoplasms, splenic B-cell lymphoma and leukemia were clearly identified and include four distinct entities: hairy cell leukemia (HCL), splenic marginal zone lymphoma (SMZL), splenic diffuse red pulp lymphoma (SDRPL) and the new entity named splenic B-cell lymphoma/leukemia with prominent nucleoli (SBLPN). The BRAF^V600E^ mutation is detected in nearly all HCL cases and offers a possibility of targeted therapy. BRAF inhibitors (BRAFi) represent effective and promising therapeutic approaches in patients with relapsed/refractory HCL. Vemurafenib and dabrafenib were assessed in clinical trials. The BRAF^V600E^ mutation is missing in SDRPL and SBLPN: mitogen-activated protein kinase 1 (MAP2K1) mutations were found in 40% of SBLPN and VH4-34+ HCL patients, making possible to use MEK inhibitors (MEKi) such as trametinib, cobimetinib or binimetinib in monotherapy or associated with BRAFi. Other mutations may be associated and other signaling pathways involved, including the B-cell receptor signaling (BCR), cell cycle, epigenetic regulation and/or chromatin remodeling. In SDRPL, cyclin D3 (CCND3) mutations were found in 24% of patients, offering the possibility of using cell cycle inhibitors. Even if new emerging drugs, particularly those involved in the epigenetic regulation, have recently been added to the therapeutic armamentarium in HCL and HCL-like disorders, purine nucleoside analogs more and more associated with anti-CD20 monoclonal antibodies, are still used in the frontline setting. Thanks to the recent discoveries in genetics and signaling pathways in HCL and HCL-like disorders, new targeted therapies have been developed, have proven their efficacy and safety in several clinical trials and become essential in real life: BRAFi, MEKi, Bruton Tyrosine Kinase inhibitors (BTKi) and anti-CD22 immunotoxins. New other drugs emerged and have to be assessed in the future. In this article, we will discuss the main mutations identified in HCL and HCL-like disorders and the signaling pathways potentially involved in the pathogenesis of the different hairy cell disorders. We will discuss the results of the recent clinical trials, which will help us to propose an algorithm useful in clinical practice and we will highlight the different new drugs that may be used in the near future.

## Introduction

The 5th edition of the World Health Organization (WHO) classification of lymphoid neoplasms recently changed (1). In the category of mature B-cell neoplasms, splenic B-cell lymphoma and leukemia were clearly identified with four distinct entities: hairy cell leukemia (HCL), splenic marginal zone lymphoma (SMZL), splenic diffuse red pulp lymphoma (SDRPL) and the new entity named splenic B-cell lymphoma/leukemia with prominent nucleoli (SBLPN).

HCL, first recognized in 1958, is a well-defined entity and a rare disease. The overall age-adjusted to the 2000 United States standard population is 0.8 per 100,000 person-years in non-Hispanic men (2) and 0.5 in women. The number of new HCL cases expected per year is 1.500 in the United States and also in Europe. The diagnosis of HCL is based on the identification in the peripheral blood (PB) and/or in the bonne marrow (BM) of hairy cells that express CD103, CD123, CD25 and CD11c. The BRAF^V600E^ mutation in the B-raf protooncogene (BRAF) was demonstrated in more than 90% of cases (3). In SMZL, blood smear examination shows a mixture of heterogeneous lymphoid cells with monocytoid and plasmacytoid differentiation (4). SMZL is also characterized by an expansion of the splenic white pulp with the infiltration of the red pulp showing a biphasic pattern. SDRPL, more recently identified in 2008 (5), is defined by a diffuse red pulp infiltration by mature B-cells effacing the white pulp. The frequency has not yet been established in the general population but SDRPL accounts for 9% of all splenic B-cell lymphomas. The small to medium-sized abnormal cells present a small or not visible nucleolus and the villous projections have a polar distribution. Some lymphoplasmacytoid cells are often observed. Monocytopenia is absent and the abnormal lymphoid cells do not express CD25 or CD123. The *BRAF^V600E^* mutation is never detected. A scoring system, based on five membrane markers (CD11c, CD22, CD76, CD27 and CD38) was proposed to distinguish SDRPL from SMZL: one point was attributed when the ratio of fluorescence intensity (RFI) CD11c was higher than 25, the CD22 RFI higher than 130, the CD76 positive, the CD27 negative and the CD38 negative. Using this scoring system, SDRPL cases scored 3 to 5 with no case scoring less than 3 whereas all available SMZL cases had a low score (0 to 2). SBLPN was recently introduced, replacing the previous term of HCL variant. The blood picture is monomorphic with large cells and prominent nucleoli and the hair-like cytoplasmic projections are circumferential. The cells usually do not express CD25 and CD123 (6).

**Figure 1:**
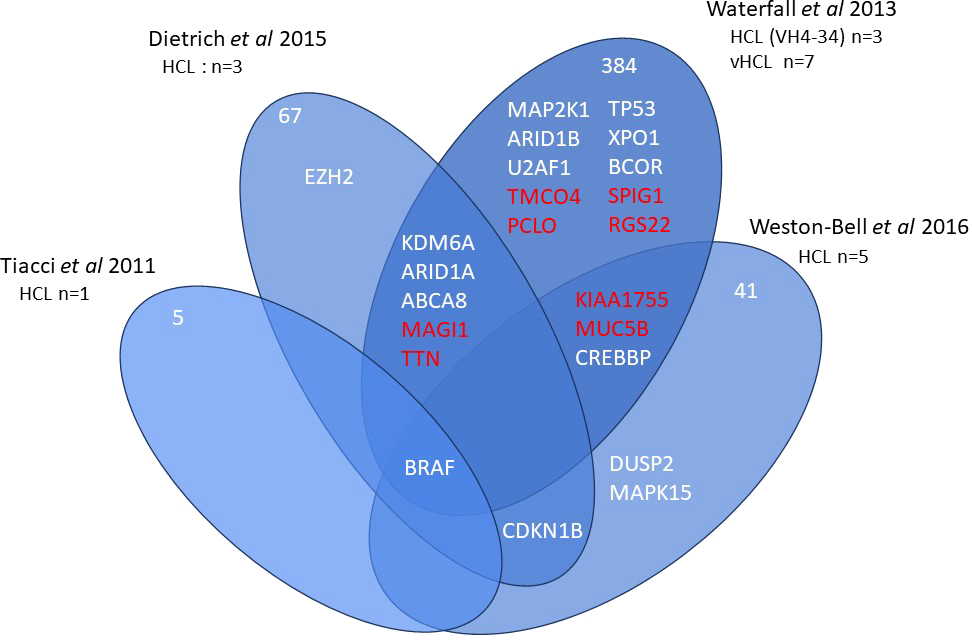
Whole exome sequencing in HCL. Venn diagram of main genes or genes of interest found in whole exome sequencing of hairy cell leukemia (HCL).

**Figure 2:**
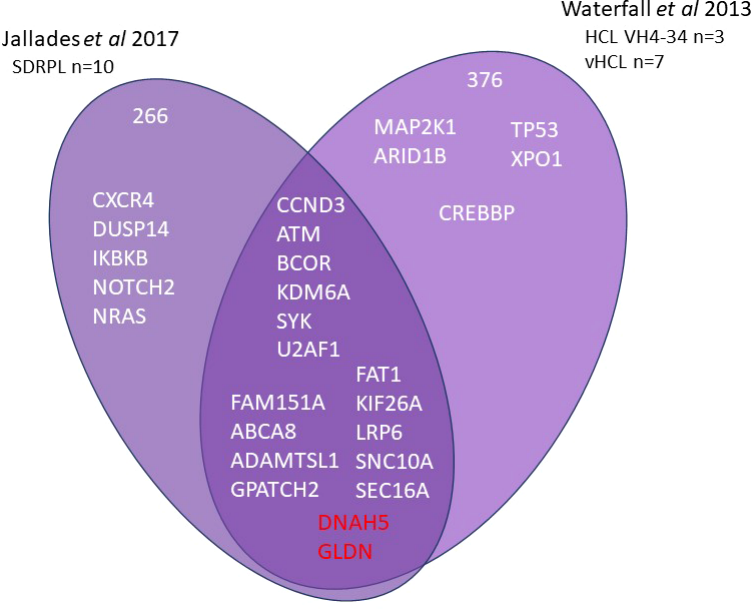
Whole exome sequencing in HCL-VH4-34, SBLPN and SDRPL Venn diagram of main genes or genes of interest found in whole exome sequencing of splenic B lymphoma/leukemia with prominent nucleoli (SBLPN), splenic diffuse red pulp lymphoma (SDRPL) and hairy cell leukemia VH4-34.

Current information on the different somatic mutations observed in the different entities is based on limited data, namely whole exome sequencing (WES) (3, 7–9) or targeted sequencing analysis (10, 11) (**Figures 1**, **2**). The activation of RAS-Mitogen-Activated Protein Kinases (MAPK) signaling is the key therapeutic target in HCL. BRAF inhibitors (BRAFi) (vemurafenib, dabrafenib, encorafenib) and also mitogen-activated protein kinase (MEK) inhibitors (MEKi) (trametinib, binimetinib) represent effective and promising therapeutic approaches in patients with relapsed/refractory HCL. However, other mutations may be associated and other signaling pathways involved: cell cycle, epigenetic regulation and/or chromatin remodeling. Receptor B-cell signaling (BCR) was also demonstrated in HCL pathogenesis. Weston Bell et al. reported that BCRs of HCL cells responded to antibody-mediated cross-linking with an increase in cellular calcium levels (12), ERK phosphorylation and apoptosis. Conversely, the ability of BCR cross–linking to protect primary HCL cells from undergoing spontaneous apoptosis was reported *in vitro*. Importantly, pretreatment with Bruton’s Tyrosine kinase (BTK) inhibitors (BTKi) completely abrogated these effects suggesting a therapeutic relevance of the BCR pathway in HCL. BTKi with ibrutinib or other BTKi recently introduced, particularly second generation BTKi, can be useful in some cases. Venetoclax, a Bcl-2 inhibitor (Bcl2i) approved for the treatment of chronic lymphocytic leukemia (CLL) is able to induce primary HCL cell apoptosis *in vitro* and could be a potential therapy for HCL (13).

The diagnosis between all these entities may be challenging: the main differences are listed in **Table 1**. In this article, we will discuss the main molecular alterations in splenic B-cell lymphomas and leukemias and new identified target drugs we can use in clinical practice allowing an effective chemotherapy-free treatment in relapsed/refractory patients.

**Table 1:** Characteristics of hairy cell leukemia (HCL) and HCL-like disorders.

	HCL	vHCL/SBLPN	SDRPL	SMZL
Incidence(100.000 inhab)	0,3	0,2	nd	0,2
Overall survival(% pour 5 years)	78-92%	57%	93%	83-91%
CLINICAL FEATURES				
Sex-ratio (M/F)	4	1.6	1.6	0.5
median age (years)	55	70	77	62
Splenomégaly	Yes	Yes	Yes	Yes
Monocytopenia	Yes	No	No	No
CYTOLOGY				
Villi	Long, fine and circumferential	Long, fine and circumferential,sometime shaggy	Long, large broad base	Small
Nucléoli	Occasional inconspicious	Constant, prominent	Occasional	Small
Chromatin	Mature, homogene	Mature, homogene	Condensed	Condensed
IMMUNOPHENOTYPE and HISTOLOGY				
Annexine A1	+	–	–	–
CD11c	+ BRIGHT	+ (63%)	+ (>90%)	+ dim (33-67%)
CD25	+ BRIGHT	- (>90%)	- (>90%)	- (78-88%)
CD103	+ BRIGHT	+ (65-100%)	- (62-84%)	- (>90%)
CD123	+ BRIGHT	- (60%)/dim	- (50-84%)	- (75%)/dim
CD27	- (>90%)	nd	- (81%)	+ (89%)
CD180	+	nd	+ FORT	+
CD200	+ BRIGHT	–	–	–
BOM (infiltration)	IS, fibrosis	IN, IS	IN, IS	IS, N
Spleen (infiltration)	PR	PR	PR	PB: nodular
CYTOGENETIC and GENETIC				
Abnormal Karyotype	40%	rare,complexe Karyotype	30%	80%
Chromosomic anomalies	del17p, +12, del7q	del17p, +12, Del7q	+3, del7q	+3, +18, del7q
IGHV mutés (%)	83-90%	46-73%	79%	59-68%
IGHV repertory (>10%)	VH3-30, VH3-23, VH4-34	VH4-34	VH4-34, VH3-23	VH1-2, VH4-34
*BRAF ^V600E^* mutation	+	–	–	–
*MAP2K1* mutations	0-18,5%	9-48%	10,50%	nd
*CKDN1B* mutations	16%	0%	nd	nd
*NOTCH1* mutations	nd	nd	10,50%	5%
*NOCH2* mutations	0%	nd	0-3%	17-21%
*TP53* mutations	0-3%	25%	0-5%	13,5-25%
*BCOR* mutation/deletion	0-5%	0%	24%	2%
*KTM2C* mutations	15%	25%	nd	nd
*CCND3* mutations	0%	13%	21-24%	13%
KLF2 mutations	10-17%	nd	nd	19-42%

vHCL, Hairy cell leukemia variant; SBLPN, Splenic B-cell lymphoma/leukemia with prominent nucleoli; SDRPL, Splenic diffuse red pulp lymphoma. SMZL, Splenic diffuse marginal zone lymphoma; HCL, Classical Hairy cell leukemia, +, positive (% of positive cases), -, negative (% of negative cases), dim, Reduced expression; IN, interstitial, IS intrasinusoidal, N, nodular RP, Red Pulp, WP, White Pulp; BM, Bone marrow. nd, Not specified.

### Mutations in splenic B-cell lymphomas and leukemias

#### Somatic mutations in hairy cell leukemia

*BRAF^V600E^* mutation is detected in nearly all HCL cases and offers a possibility of targeted therapy. By using WES of genomic DNA from purified leukemic cells of one HCL patient, the mutation V600E of the *BRAF* gene (*BRAF^V600E^*) (7q34) as well as *CSMD3*, *SLC5A1*, *CNTN6* and *OR8J1* mutations were identified in 2011 (3). *BRAF^V600E^* was detected in 48 other HCL patients by Sanger sequencing and was not found in 195 patients with other B-cell chronic lymphoproliferative disorders (B-CLPD), suggesting *BRAF^V600E^* mutation could be an early and genetic driver in HCL. The mutation replaces thymine (T) with adenine (A) in exon 15 of BRAF at position 1799 of the gene coding sequence. This substitution produces an amino acid change from valine (V) to glutamate (E) at position 600 (V600E) of the protein sequence and leads to aberrant activation of the protein serine threonine kinase B-raf. The *BRAF^V600E^* mutation activates the mitogen-activated protein kinases extracellular signal regulated kinases (MEK-ERK) pathway, leading to enhanced cell proliferation, survival, and ultimately neoplastic transformation.

*BRAF^V600E^* mutation is also detected in solid tumors with a high incidence of 80% in cutaneous melanoma. Benign naevi of the skin are BRAF^V600E^ while they can stay indolent for many years (14). It is detected in 50% of Langerhans histiocytosis (LCH), and Erdheim Chester disease (ECD) and much more rarely in lung, ovarian, bladder, thyroid, prostatic cancers, cholangiocarcinoma or sarcoma/GST. The mutation was also identified in other B-CLPD, including CLL and multiple myeloma (MM) in less than 5% of cases.

Alternative *BRAF* mutations in exon 11 should be excluded in patients without *BRAF^V600E^* mutation. In 24 HCL and 194 various mature B or T-cell neoplasms, *BRAF* mutations were investigated in exon 11 (15). All non-HCL lymphomas lacked *BRAF* mutations. Twenty-one HCL patients carried the BRAF^V600E^ mutation in exon 15. In addition, two patients presented BRAF mutation in exon 11 (F468C, D449E). The last patient presented a *BRAF^V600E^* mutation associated with a S602T mutation in exon 15 of B-raf. Two patients showed wild-type sequences for *KRAS* (exons 2, 3, 4), NRAS (exons 2,3,4) and *HRAS* (exons 2,3). In the last patient, wild type sequences were found in KRAS (exons 2,3,4) *NRAS* (exons 2,3) and *HRAS* (exon 2). The possible and functional consequences of the mutations in exon 11 were not analyzed.

In patients with wild type *BRAF* (*BRAF^WT^*), the need for developing new therapeutic targets is crucial. Ten per cent of HCL cases are *BRAF^WT^*. As in CLL, the immunoglobulin variable heavy chain (IGHV) rearrangements have a clinical impact. Patients with an unmutated (UM) IGHV profile have shorter overall survival (OS) than those with a mutated (M) profile. These patients and 40% of patients with SBLPN, use IGHV4-34 rearrangements (VH4-34^+^). VH4-34^+^ HCL cases could represent a subset of HCL associated with a poor prognosis: higher disease burden at diagnosis, poor response to standard therapy, shorter overall survival (OS) and absence of *BRAF^V600E^* mutation (16, 17).

Additional somatic mutations could play a role in the progression of the disease. When using WES in 3 patients, who were refractory to purine nucleoside analogs (PNAs) and who received vemurafenib, two patients presented mutations of cyclin dependent kinase inhibitor 1 B (*CDKN1B*, p27) (12p13). p27 protein regulates the transition from G1 to S phase of the cell cycle. Targeted deep sequencing including *CDKN1B* (exons 1,2) and *BRAF^V600E^* (exon 15) in a larger cohort of 81 patients showed *BRAF^V600E^* in all patients and deleterious CDKN1B mutations in 13/81 patients (16%) (8). In 11 patients, the allele frequencies were very similar to those observed in the BRAF mutant clone, suggesting that *CDKN1B* mutations could be also an early event playing a role in the pathogenesis of HCL. In those cases, CDK inhibitors (CDKi) could be an option.

When performing WES in 5 HCL cases (9), 63 novel nonsynonymous somatic variants (SVs) were identified in the exomes including dual specificity phosphatase 2 (*DUSP2*) (2q11.2) encoding an inhibitor of ERK in MAPK signaling. Other SVs were identified such as *CHD7*, *SLC2A8* and *CLE6A* with allele frequencies comparable to *BRAF* and could be potential drivers. In a large cohort of 98 patients, inactivating Kruppel like factor (*KLF2*) (19p13.1) mutations were the second most altered genes after *BRAF* and observed in 21% of cases (11). *KLF2* is a transcription factor controlling the differentiation of multiple B-cell subpopulations, including marginal zone B-cells. Mutations of the genes of the epigenetic regulation were frequently observed, with mutations in EZH2 (7q36.1), the histone methyltransferase *KMT2C* (MLL3) (7q36.1) occurring in 15% of patients and more rarely in histone demethylase *KDM6A* (UTX) (Xp11.3) or histone acetyltransferase *CREBPP* (CBP) (16p13.3). Other mutations in the chromatin remodeling complex family *ARID1A* (1p36.11) and *ARID1B* (6q25.3) were also described.

#### Somatic mutations in splenic marginal zone lymphoma

Most recurrent mutations are *NOTCH2* (1p12) (10-25% of cases), *KLF2* (20-30% of cases), *TP53* (17p13.1) (10-15%), mutations in the NF kappa pathway such as *MYD88* (3p22.2) (5-15%) and also mutations of the epigenetic regulation (4).

#### Somatic mutations in splenic diffuse red pulp lymphoma

The *BRAF* ^V600E^ mutation is missing in SDRPL. Cyclin D3 (*CCND3*) (6p21.1) mutations were found in 24% (6/25) of patients with SDRPL, as well as recurrent mutations or losses in *BCOR* (gene encoding the *BCL6* corepressor (Xp11.4) (18). When using immunostaining in 37 splenectomy specimens, 24 showed cyclin D3 expression in > 50% of cells and 9 cases had a lower level. In contrast, when investigating 74 SMZL, 35 mantle cell lymphoma (MCL), 7 HCL and 40 CLL, cyclin D3 was considered to be positive in only 4 patients (1 SMZL, 1 HCL and 2 MCL) (19). In a series of 19 SDRPL patients including 5 patients with progressive disease, 4 patients presented mutations: *NOTCH1* mutations in 2 cases, *TP53* mutations or *MAP2K1* mutations in one case, respectively (20). *KLF2* mutations were rarely described in SDRPL.

#### Somatic mutations in splenic B-cell lymphoma/leukemia with prominent nucleoli

Mitogen-activated protein kinase 1 (*MAP2K1*) (15q22.1-q22.3) mutations are found in 40% of SBLPN. The frequency of *MAP2K1* mutations is twice as high in SBLPN as compared to that observed in HCL. *MAP2K1* mutations are identified in VH4-34^+^ HCL patients. When using WES, activating mutations in *MAP2K1* gene were identified in 5/10 samples, including 2 VH4-34^+^ HCL and 3 VH4-34^-^ HCL. In a validation set of 21 additional samples (4 HCL, 17 SBLPN), Sanger sequencing identified 10 other positive samples: 3 VH4-34^+^ HCL and 7 SBLPN (3 IGHV4-34^-^, 4 IGHV4-34^+^). In the last set of IGHV4-34^-^ HCL patients, just one patient presented *MAP2K1* mutation. Out of 51 patients (27 HCL, 24 SBLPN), *MAP2K1* mutations were observed in exons 2 and 3 in 16/51 patients (31%): 6 HCL (5 VH4-34^+^, 1 VH4-34^-^), 10 SBLPN (6 VH4-34^-^, 4 VH4-34^+^). All but one of the mutations were substitutions (C121S being the most frequent: 3/16 patients). One SBLPN patient presented a forty-eight nucleotide in frame deletion. *MAP2K1* mutations were detected in 6/27 HCL patients (22%), including 5 VH4-34^+^ HCL and in 10/24 (42%) SBLPN, including 4 IGHV4-34^+^ SBLPN. In case of *BRAF^WT^* gene, mutations in *MAP2K1* encoding MEK (downstream protein of B-raf) were found in more than half of the cases. The *MAP2K1* mutations also activate the MAPK pathway. Note that some mutations allow the use of MEKi, while others involving the binding site of the inhibitor can lead to resistance. The frequency of *MAP2K1* mutations is much lower in SDRPL, with an estimated frequency of 7% (18). Recurrent hotspot mutations of U2 Small Nuclear RNA Auxiliary Factor 1 (*U2AF1*) encoding a protein belonging to the spliceosome were detected in 15% of SBLPN. While no *TP53* mutations were frequently observed in HCL, *TP53* aberrations accounting for 30-40% of SBLPN are associated with a significant risk for resistance to chemotherapy (10).

### Signaling pathways in HCL

#### RAS-BRAF-MEK-ERK signaling pathway

**Figure 3:**
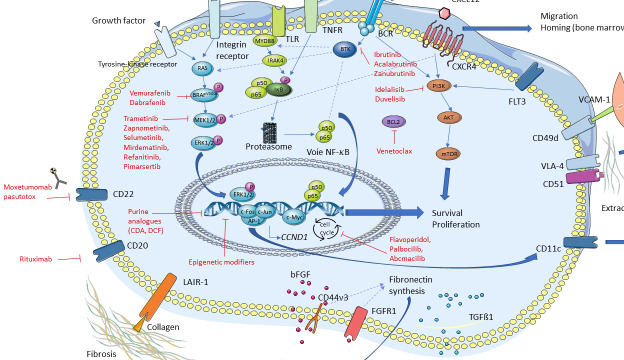
Signaling pathways in hairy cell leukemia (HCL). In red: drugs of targetable pathways.

Major discoveries have been recently made in HCL and HCL-like disorders, particularly regarding the importance of several signaling pathways in the pathophysiology of these diseases (**Figure 3**). As previously mentioned, the *BRAF^V600E^* mutation is frequent in HCL, thus leading to a constitutive activation of the RAS-BRAF-MEK-ERK signaling pathway. Activation of this axis is responsible for the typical hairy morphology, immunophenotype and clinical presentation found in HCL. This signaling pathway may be targeted by specific drugs: BRAFi like vemurafenib, dabrafenib and encorafenib; and MEKi like trametinib and binimetinib (21–25).

#### BCR and CXCR4 signaling pathways

More recently, the major role of the BCR pathway has been highlighted in HCL. The BCR complex is made up of an Ig associated with CD79a and CD79b whose cytoplasmic tails contain immunotyrosine-based activation motifs (ITAM). In normal and malignant B-cells, BCR cross-linking by antigens is responsible for the activation of a phosphorylation cascade involving several kinases and adapter proteins like Src kinases, spleen tyrosine kinase (SYK), BTK, phospholipase C gamma 2 (PLCγ2), leading to intracellular calcium mobilization and activation of MAPK and nuclear factor kappa B (NF-κB). Activation of this cascade induces B-cell survival, proliferation, migration, resistance to apoptosis, and secretion of chemokines like C-C motif ligand 3 (CCL3) and CCL4 (26, 27). This secretion of CCL3 and CCL4 attracts T-cells and monocytes.

In B-cell malignancies, the role of the BCR signaling pathway was proven in the pathophysiology of CLL (27), Waldenström macroglobulinemia (28), diffuse large B-cell lymphoma of the activated B-cell subtype (DLBCL-ABC) (29), mantle-cell lymphoma (MCL) (30), MZL (31), and more recently HCL and SBLPN. The mechanisms of activation of the BCR vary according to the B-cell malignancy: BCR stimulation by foreign or self-antigens, mutations in components of the BCR pathway resulting in autonomous or enhanced antigen-induced BCR signaling (i.e., tonic BCR).

From a functional point of view, *Weston-Bell and colleagues* observed that in HCL, BCR cross-linking leads to an increased intracellular calcium level, ERK phosphorylation and apoptosis (12). On the contrary, *Sivina et al.* demonstrated that BCR cross-linking with anti-IgA/IgG/IgM improves HCL cells viability. They also demonstrated that BCR cross-linking increases the phosphorylation of BTK, ERK and AKT, and the secretion of CCL3 and CCL4 by hairy cells (32). In this BCR pathway, BTK plays a major role and can be targeted by BTK inhibitors (BTKi), already used in CLL (33), MCL (30), MZL (31), Waldenström macroglobulinemia (28), and DLBCL-ABC (29). Of note, HCL cells express different isotypes of BCR. Thus, the response of HCL cells to BCR stimulation is heterogenous (34).

C-X-C motif chemokine receptor 4 (CXCR4, CD184) is the receptor of the chemokine C-X-C motif ligand 12 (CXCL12). HCL cells express high levels of CXCR4 on their membrane surface and CXCL12 is secreted by bone marrow stromal cells (BMSC). Thus, the cross-talk between CXCR4 and CXCL12 leads to the migration of HCL cells in the bone marrow (BM), explaining the frequent involvement of BM in HCL (35–37). The importance of the interactions between HCL cells and their tumor microenvironment has been reviewed elsewhere (38, 39). CXCR4 cross-linking leads to the activation of a signaling pathway, and BTK is also a fundamental signal transducer in this CXCR4 pathway, both in normal and in CLL B-cells (33, 40, 41).

Ibrutinib is an oral, first-in class, and irreversible BTKi. Ibrutinib is used in CLL and other B-cell malignancies. Given the role of the BCR and CXCR4 pathways in HCL, and the importance of BTK in these pathways, ibrutinib was tested in preclinical experiments using HCL cell lines and primary HCL cells (32). The BCR of these cells was stimulated with anti IgA/IgG/IgM. Pre-incubation with ibrutinib reduced HCL cell survival, BTK phosphorylation, downstream kinases phosphorylation and secretion of CCL3 and CCL4. After CXCR4 stimulation of HCL cells using CXCL12, pre-incubation with ibrutinib reduced ERK phosphorylation. These experiments highlight the importance of the BCR and CXCR4 signaling pathways in HCL cells survival and open the way to the clinical use of BTKi in HCL (see below). BRAF is an important mediator of the BCR signaling pathway and the activation of MAPK (42). Interestingly, the BRAF^V600E^ mutation has no impact on response of B-cells to BCR stimulation (32, 34).

The phosphoinositide 3-kinase δ (PI3Kδ), which is linked to the BCR pathway, seems to play a role in the pathophysiology of HCL as well (43).

Therefore, several signaling pathways play a major role in the pathophysiology of HCL and HCL-like disorders, especially the BRAF, BCR and CXCR4 pathways. These recent discoveries open the way to the use of targeted therapies in these hematological malignancies.

### New targeted drugs in HCL and HCL-like disorders

The potential new drugs we can use in clinical practice are listed in **Figure 3** and **Table 2**.

**Table 2:** Drugs currently used in hairy cell leukemia (HCL) and HCL-like disorders.

Drugs	n	ORR (%)	CR (%)	uMRD (%)	PFS	OS	Relapses	Adverse events	Follow-up	FDA approved	EMA approved	Study
**Cladribine**	159	99	83	-	median 163 months	97% at 5 years	31%	myelosuppression,immunosuppression, rash	116 months	Yes	Yes	*Paillassa et al*
**Pentostatin**	49	100	84	–	median 159 months	86% at 5 years	29%	myelosuppression,immunosuppression, rash	126 months	Yes	No	*Paillassa et al*
**Rituximab + Cladribine**	34*	100*	100*	97*	-	-	0%*	myelosuppression,immunosuppression, rash	96 months	No	No	*Chihara et al*
**Vemurafenib**	50	96-100	35-42	–	73% at 1 year**	91% at 1 year**	27% at 1 year**	cutaneous, articular, ocular, hepatic, pancreatic, QT intervalprolongation	11,7 months**	No	No	*Tiacci et al*
**Dabrafenib**	10	80	30	0	10%	90%	88%	cutaneous, articular, ocular,hepatic, pancreatic, QT interval prolongation	64 months	No	No	*Tiacci et al*
**Dabrafenib + Trametinib**	55	89	66	25% of CR pts	94% at 24 months	95% at 24 months	6%	cutaneous, articular, ocular, pyrexia, neutropenia, hyperglycemia, hepatic	43 months	No	No	*Kreitman et al*
**Rituximab + Vemurafenib**	30	87	87	65% of CR pts	78%	-	15%	cutaneous, articular, ocular, hepatic, pancreatic, QT intervalprolongation	37 months	No	No	*Tiacci et al*
**Ibrutinib**	37	54***	19***	–	73% at 36 months	85% at 36 months	–	bruising, hypertension, atrial fibrillation/flutter, cytopenias, diarrhea, nausea, fatigue, respiratory infection, myalgias	3,5 years	No	No	*Rogers et al*
**Moxetumomab pasudotox**	80	75	41	82% of CR pts	median 72 months	-	-	capillary leak syndrome, hemolytic and uremic syndrome, pyrexia, nausea, peripheral edema, headache	24,6 months	Yes	Withdrawn	*Kreitman et al*

*Outcomes in the CDAR group (i.e. CDA with simultaneous R). **In the US trial. ***As best response at any time.

#### PNA with or without rituximab: The gold standard in first line

It is important to highlight that for patients without any of the following criteria, a ‘watch and wait’ strategy is preferred: hemoglobin < 11 g/dL, platelet < 100 G/L, absolute neutrophil count (ANC) < 1 G/L, symptomatic organomegaly, recurrent infections, or constitutional symptoms (44, 45).

Even if new drugs have recently been added to the therapeutic armamentarium in HCL and HCL-like disorders, conventional chemotherapies are still used in the frontline setting. According to the most recent NCCN guidelines, cladribine (CDA) with or without rituximab (R) or pentostatin (DCF) are recommended in first line (44). Currently, there is no recommendation to prefer one PNA over another. In recent multicenter cohorts with a long-term follow-up over 10 years, outcomes for HCL patients treated with PNA were impressive. Thus, in the French cohort (46), for patients treated with PNA in first line for HCL, the overall response rate (ORR) was 100% and the complete response (CR) rate 83%. The median relapse-free survival after PNAs in first line (RFS1) was 163 months for patients treated with CDA and 159 months for those treated with DCF. The 5-year overall survival (OS) was 97% and 86% for patients who received CDA and DCF, respectively. In an Italian multicenter study (47) including 513 patients treated with CDA as first line therapy, the ORR and CR rates were 91.8% and 65.3%, respectively. The median RFS was 12.2 years and the 5-years OS 95.3%.

Recently, the combination of CDA with R was shown to be very effective with undetectable measurable residual disease (uMRD) even in high-risk patients like those with vHCL, who are usually less responsive to PNA monotherapy. The treatment schedule was variable between the different studies: frequency and number of R infusions, sequential or simultaneous schedule, R after PNA for if MRD was detectable (dMRD). In a first phase 2 study, 80 patients including 59 treatment-naïve patients received CDA followed by eight weekly doses of R 1 month later. The CR rate was 100%, the 5-year progressive-free survival (PFS) and OS were 94.8% and 96.8%, respectively (48). In a second phase 2 study, HCL patients were randomized between two groups: CDA with simultaneous R (i.e. R started at day 1 of CDA, eight weekly doses of R, CDAR group) or CDA with delayed R (i.e. R started 6 months later, eight weekly doses of R, CDA group) (49). At 6 months, the CR rate was 100% for CDAR *versus* 88% for CDA, and the CR rate with uMRD was 97% for CDAR *versus* only 24% for CDA. The CDAR regimen was also effective in vHCL: in 20 patients (including 8 previously untreated) treated with CDAR (50), the CR rate at 6 months was 18/20 (90%) with 16 uMRD (80%) (MRD measured with flow cytometry (FCM) and immunohistochemistry (IHC) in peripheral blood and BM). The 5-years PFS and OS were 63.3% and 74%, respectively. The patients with dMRD after at least 6 months after initial therapy received a second course of eight weekly doses of R. Eleven patients received a second course of R between 6 and 82 months after initial treatment, and two additional patients received a second chemoimmunotherapy because of a rapid progressive disease. The patients who achieved uMRD at 6 months had a longer PFS and OS compared with patients with dMRD: not reached *versus* 17.4 months, not reached *versus* 38.2 months, respectively. Moreover, the 5/19 evaluable patients harboring a TP53 mutation had more dMRD.

Bendamustine is another PNA that has been shown to give some good results in HCL and vHCL, in combination with anti-CD20 monoclonal antibodies (51). In a prospective study in which 12 patients with R/R HCL received bendamustine + R, all patients achieved response, 7/12 CR, and 6/12 CR with uMRD (using FCM in BM). The 6 patients achieving CR with uMRD were still in CR at a median follow up of 31 months (52). In vHCL, patients achieved CR after treatment with bendamustine + R in first line or in relapse (53, 54). The association of bendamustine and obinutuzumab was effective in a 48 year old woman with R/R HCL, achieving a CR with uMRD (FMC in BM) (55). A phase 2 clinical trial in which patients with multiply relapsed or refractory HCL are randomized between bendamustine + R or pentostatin + R is currently recruiting.

Thus, the combination of PNA with anti-CD20 monoclonal antibodies give durable CR with uMRD. These combinations are particularly useful in SBLPN and this is our preferred choice in this highly refractory population. Even if PNA with or without R are effective, most patients relapse and become less and less sensitive to these conventional chemotherapies, and the duration of response (DOR) is shorter at each relapse (46, 56). Although responses are frequent in first relapse, especially when R is added, some patients become refractory to PNA. Moreover, toxicities of PNA are a major concern. Indeed, PNA are responsible for a high level of myelosuppression, with frequent cytopenia. PNA are also immunosuppressive, with a significant risk of opportunistic infections in this high-risk population. HCL patients treated with PNA should be considered immunocompromised for several years: after a treatment with cladribine, number and function of NK cells and CD8^+^ T-cells normalize within 2-3 months, monocytopenia and number of dendritic cells more than 1 year, number of B-cells within 1-2 years, and number of CD4^+^ T-cells within 2-5 years. This quantitative and qualitative defect in immune cells partly explains the risk of second neoplasms. Patients treated with PNA, in particular when drugs like allopurinol and/or cotrimoxazole are co-administered, often develop a cutaneous rash. These frequent side effects, especially infectious diseases and second cancers, associated with the need for hospitalization, make the use of PNA expensive (46, 57–68). Therefore, alternative and targeted therapeutic strategies are needed for these patients.

#### BRAFi and MEKi

The importance of the RAS-BRAF-MEK-ERK signaling pathway due to mutations of *BRAF* in HCL and *MAP2K1*, encoding MEK, in SBLPN, led to the development of trials with BRAFi like vemurafenib, dabrafenib or encorafenib, and/or MEKi like trametinib or binimetinib in relapsed/refractory (R/R) HCL and HCL-like disorders.

In two phase 2 studies (Italy and US) enrolling 54 patients with R/R HCL, vemurafenib was administered at 960 mg twice daily for 16-18 weeks (69). The ORR was 96% in the Italian study, 100% in the US study, and the CR rate 35% and 42%, respectively. In the Italian study with a median follow-up of 23 months, the RFS1 was 9 months: 19 months for patients achieving a CR and only 6 months for those achieving PR. In the US study, the 1-year RFS and OS were 73% and 91%, respectively. The results of the US study were recently updated: after a median follow up no longer of 11.7 months but of 40 months, the median RFS1 was 19 months without significant difference between CR and PR patients. In patients retreated with vemurafenib, the RFS2 was 12.7 months and was not statistically different from RFS1 (70).

Lower doses of vemurafenib may be effective in HCL. *Dietrich et al.* analyzed 21 R/R HCL patients, who received vemurafenib at individual dosing regimen outside clinical trials (240-1920 mg/d) (71). The median duration of treatment was 90 days. The CR rate was 40% (6/15 evaluable patients) and the median event-free survival (EFS) was 17 months. The response rate and the side effects were independent of the dose and duration of treatment. Moreover, for patients who relapsed after a first course of vemurafenib, re-treatment with vemurafenib led to responses allowing stop and go strategies. The most frequent adverse events attributable to vemurafenib were: arthralgia, arthritis, rash, photosensitivity, basal-cell carcinoma, squamous-cell carcinoma, melanoma, ocular toxicity, QT interval prolongation, transaminitis, elevation of pancreatic enzymes. Adverse events often required dose reductions (69). The drug is given orally, so it presents practical aspects for patients. Another advantage is the absence of myelotoxicity of vemurafenib. Thus, contrary to PNA, it can be administered in HCL patients who have an active infection and who cannot wait for the resolution of this infection to start treatment against HCL. In a publication by *Bohn et al.*, off-label low-dose vemurafenib (480-960 mg/d) was given to 6 R/R HCL patients with an active infection (72). In all patients (including one patient with an invasive pulmonary aspergillosis and another one with a septic shock and multi-organ failure), vemurafenib allowed rapid neutrophil count recovery and resolution of infection. Two patients achieved a CR and 4 patients a PR, resulting in an ORR of 100%. Interestingly, 2 patients tolerated vemurafenib well whereas they received the drug without a fixed duration (for 32 and 41 months at last follow-up) with serial dermatologic follow-up. Moreover, after achieving PR with vemurafenib and resolution of pneumonia, a patient started a consolidation with CDA. Thanks to the absence of myelosuppressive effect, BRAFi like vemurafenib might be an option to treat HCL patients during the COVID-19 pandemic, even in first line (73). BRAFi are currently not approved in HCL, are used ‘off-label’, and the duration of response is still short in monotherapy, as previously shown, so they might be used as a bridge to PNA during COVID-19 waves.

Dabrafenib, another BRAFi, was also evaluated in R/R HCL. In a pilot single-center phase 2 trial enrolling 10 patients with R/R HCL (median age: 62 years old, median previous lines: 3.5), dabrafenib was given at 150 mg twice daily for 12 weeks, except for one patient who received the drug for 8 weeks because of rapid CR achievement (25). The ORR was 80%: CR in 3/10, PR in 5/10, minimal response (MR) in 2/10. Interestingly, 2 patients had previously been treated with vemurafenib: 1 reached CR and 1 PR after dabrafenib. No uMRD was observed. Blood cell counts quickly improved after starting treatment. At a median follow-up of 64 months, the PFS was 10% and the OS was 90%. The toxicity profile of dabrafenib was similar to that of vemurafenib, with dose reduction in 6/10 patients. Adverse events were mainly low grade and manageable. *Kreitman et al.* presented the results of a phase 2 basket study including a cohort of 55 R/R HCL patients, who continuously received dabrafenib 150 mg twice daily + trametinib 2 mg once daily until disease progression or unacceptable toxicity (74). Patients were heavily pre-treated. The median patient follow-up was 43.2 months. The ORR and CR rates were 89.0% and 65.5%, respectively. 9/36 patients were uMRD (evaluation of MRD with FCM in BM and peripheral blood) and 27 patients dMRD. The 24-month PFS and OS were 94.4% and 94.5%, respectively. However, toxicities were important with 35 patients (63.6%) experiencing grade 3-4 adverse events (especially hyperglycemia, pyrexia, pneumonia and neutropenia).

The combination of BRAFi with anti-CD20 monoclonal antibodies represents a promising therapeutic strategy. In a single-center phase 2 trial, *Tiacci et al.* demonstrated the impressive efficacy and good tolerance of vemurafenib + R in R/R HCL (75). Vemurafenib was given at 960 mg twice daily for 8 weeks, and concurrent then sequential R at 375 mg/m^2^ for 8 infusions over 18 weeks to 30 R/R HCL patients. The median age was 61 years old; the median number of prior lines was 3. The ORR and CR rate were 87%. Interestingly, 65% of CR patients achieved uMRD (evaluation of MRD with PCR *BRAF^V600E^* in BM and peripheral blood) and all patients (n=7) who had previously received BRAFi alone responded to this combination. After a median follow-up of 37 months, the median PFS was 78%. uMRD and no previous BRAFi treatment were associated with longer RFS. There was no unexpected toxicity signal with no myelotoxicity. Even if it is not allowed to compare the results of two different clinical trials, these outcomes seem better than those achieved with vemurafenib monotherapy (69). Indeed, uMRD was rarely achieved and DOR was shorter with BRAFi or MEKi monotherapy.

#### BCRi

Ibrutinib was clinically evaluated in 37 HCL or SBLPN. Ibrutinib in monotherapy was prescribed at a dose of 420 mg/d (n = 24) or 840 mg/d (n = 13) until disease progression or unacceptable toxicity (76). Patients had R/R HCL (n = 28), previously untreated vHCL (n = 2), or R/R vHCL (n = 7). The median number of prior lines was 4 (range 0-12), all R/R patients had been previously treated with PNA. The ORR (CR + PR) at 32 weeks was the primary endpoint and was 24%, increasing to 36% at 48 weeks and to 54% at any time. At 32 weeks, 1 patient had CR, 8 PR, 21 stable disease (SD), 3 progressive disease (PD), and 4 were not evaluable for response. The best response at any time was CR in 7 patients (3 patients with uMRD CR), PR in 13 patients, and SD in 10 patients. The median follow-up was 3.5 years. The estimated 36-month PFS and OS were 73% and 85%, respectively. Even if a comparison between clinical trials is not statistically pertinent, these results are clearly inferior to those seen with BRAFi, MEKi, and Moxetumomab pasudotox (Moxe). In terms of adverse events, among other toxicities, bruising was seen in 43% of patients (no grade ≥ 3), atrial fibrillation in 16% (no grade ≥ 3), atrial flutter in 5% (grade ≥ 3 in 3%), hypertension in 43% (grade ≥ 3 in 11%), and heart failure in 3% (all grade ≥ 3). This toxicity profile was similar to that seen in other B-cell malignancies (30, 77).

Interesting pharmacodynamic data were brought by this study (76). Mutational analyses were realized in 4 patients with progressive disease (2 HCL, 2 SBLPN). No mutation in BTK or in PCLγ2 was found. Even if the number of patients included in this genetic analysis is small and prevents any conclusion, the mechanisms of resistance to BTKi could be different in HCL and in CLL in which mutations in BTK and in PCLγ2 have been documented in patients with CLL progressing after ibrutinib treatment (78). An analysis of expression of phosphorylated ERK (pERK) was done by IHC in HCL cells. ERK is downstream of BTK in BCR signaling, pERK is therefore a marker of BCR pathway activation, and is decreased in CLL cells treated with ibrutinib (79). This does not seem to be the case in HCL because a persistence of pERK was seen in several HCL patients after treatment with ibrutinib, and it was observed that some patients had a durable benefit from ibrutinib whereas pERK was detected after 32 and 48 weeks of treatment. The persistence of pERK in HCL cells was not associated with shorter PFS. Moreover, unlike in CLL (a phenomenon called ‘partial response with lymphocytosis’) (80), in the majority of patients, the mobilization of leukemic cells in the peripheral blood was not observed in HCL. Indeed, HCL is more related to an ‘aleukemic’ leukemia with few nodal involvement that could limit the nodal release described in CLL. Therefore, the mechanism of the effect of ibrutinib could be different between CLL and HCL and should be clarified in further studies.

Interesting data were observed for vHCL patients (76). Even if the study was not designed to compare outcomes of HCL *versus* vHCL patients, with a small number of patients for each entity, the ORR was not significantly different between HCL and vHCL patients: 54% *versus* 56%, respectively. In HCL, 6/28 (21%) patients achieved CR *versus* 1/9 (11%) patients in vHCL. There was no significant difference in PFS and OS between HCL and vHCL or between 420 mg/d and 840 mg/d. Two other publications describe the effectiveness of ibrutinib in few vHCL patients. In the first one, a patient with R/R vHCL was treated with ibrutinib continuously at 420 mg/d (81). Because of persistent thrombocytopenia, he did not meet criteria for PR but he experienced a decrease in spleen size and lymphocytosis, an improvement of hemoglobin level, and a resolution of constitutional symptoms. He had no major toxicity and was still receiving the drug after 16 months of follow-up. In the second one, two patients with R/R vHCL experienced a clinical benefit after treatment with ibrutinib 560 mg/d (82). One patient achieved a PR at 6 months, with a DOR of 16.5 months. The other patient prematurely stopped ibrutinib because of bruising and gastrointestinal toxicities but reduction of splenomegaly was observed after 3 months of treatment.

Regarding other BCRi, although they are increasingly studied/approved in CLL and other B-CLPD, to our knowledge they have not been tested in HCL or HCL-like disorders. This is the case for other BTKi like acalabrutinib, zanubrutinib, tirabrutinib, and pirtobrutinib (83, 84), and for PI3Ki like idelalisib, duvelisib, copanlisib, umbralisib, buparlisib, acalisib, and parsaclisib (85, 86). It could be interesting to evaluate efficacy and safety of these drugs in relapsed/refractory HCL. However, because of concerns about their toxicity, PI3Ki were withdrawn from the market.

#### Moxetumomab pasudotox: An immunotoxin targeting CD22

CD22, a sialic acid binding immunoglobulin-like lectin (siglec), which inhibits BCR calcium signaling, is a B-cell marker which is highly expressed on HCL cells (87). Moxetumomab pasudotox (Moxe) is the variable region of an anti-CD22 monoclonal antibody conjugated to the PE38 exotoxin of *Pseudomonas aeruginosa (* 88). In the multicenter, open-label, pivotal trial, 80 R/R HCL patients received Moxe at 40 µg/kg on days 1, 3 and 5 for a maximum of 6 28-day cycles (89), (90). Patients had at least 2 previous systemic lines of treatment. The median follow-up was 24.6 months in the updated analysis. The ORR, CR rate and durable CR rate (i.e., CR lasting more than 180 days, primary endpoint of the study) were 75%, 41% and 36%, respectively. Interestingly, 82% of CR patients reached uMRD (assessed using IHC on BM samples). Such deep responses had already been observed in the phase 1 study of Moxe in HCL (91), (92). The median PFS was 71.7 months. Of note, patients with splenomegaly or previous splenectomy had less responses to Moxe. Moreover, three patients with vHCL were included in the trial, but any of them achieved a CR. Due to the small number of patients with vHCL, no conclusions can be drawn about the use of Moxe in vHCL. Regarding toxicities, Moxe was overall well tolerated, even if capillary leak syndromes (9%, grade 3-4 3%) and hemolytic and uremic syndromes (8%, grade 3-4 5%) were observed. These adverse events were all reversible and manageable. Most other frequent adverse events were nausea (28%), pyrexia (20%), peripheral edema (26%), and headache (21%). Interestingly, Moxe is not myelosuppressive and could represent a good therapeutic option in patients at high risk of opportunistic infections (90). Moxe was approved by the FDA and the EMA in HCL patients after at least 2 previous lines including 1 PNA. However, the drug is no longer available in Europe due to development stop.

## Conclusion

Chemotherapy with PNA monotherapy or associated with anti-CD20 stay the gold standard in the frontline setting. Thanks to the recent discoveries in genetics and signaling pathways in HCL and HCL-like disorders, new targeted therapies have been developed and have proven their efficacy and safety in several clinical trials: BRAFi, MEKi, BTKi and/or anti-CD22 immunotoxins. The combination of vemurafenib with R is promising. Outcomes with ibrutinib are less impressive and Moxe is currently not available in Europe.

We encourage inclusion in clinical trials and in national and international registries for collecting real-life data, and development of e-platforms to solicit HCL experts in order to help clinicians in complex situations, as it is in progress in France. Due to the absence of myelosuppressive effects, BRAFi and MEKi could be used for patients not eligible for chemotherapy because of age, comorbidities, or active infection.

**Figure 4:**
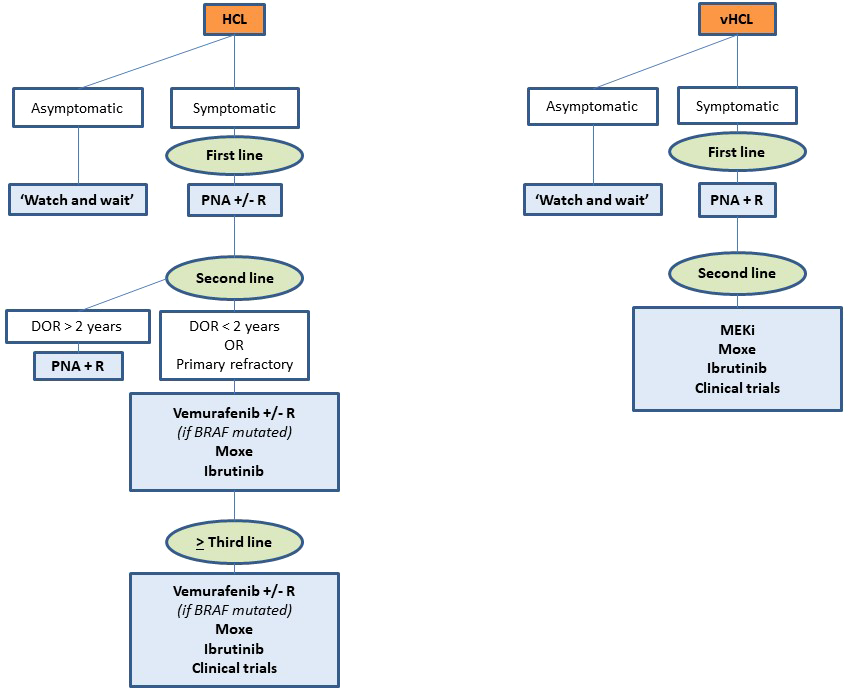
Current therapeutic algorithm in hairy cell leukemia (HCL).

The choice between all these drugs depends on many factors: number and type of prior therapies, duration and depth of previous responses, tolerance to prior treatments, age and comorbidities, patient’s preferences, costs, availability of the drugs, histologic subtype, genetic profile (*BRAF^V600E^versus BRAF* wild-type, *TP53* mutation, IGHV), and pandemic context. **Figure 4** presents our current therapeutic algorithm. In asymptomatic patients without significant cytopenia, we recommend a ‘watch and wait’ strategy. If a treatment is indicated, and if the patient is eligible for chemotherapy, we use PNA +/- R in first line. We encourage the combination with R in young and high-risk patients: SBLPN, IGHV4-34^+^ cases, unmutated IGHV or *TP53* alterations. In first relapse, if the DOR is > 2 years and if the patient is still eligible for chemotherapy, we use PNA + R. However, for patients with a DOR < 2 years, with primary refractory disease, or in second or more relapse, we recommend using targeted therapies: BRAFi +/- R (if BRAF^V600E^ mutated), Moxe or BTKi. For patients not eligible to chemotherapy, we recommend using targeted therapies in first line. In case of active infection, BRAFi or MEKi can serve as a bridge to PNA until resolution of infection. We also encourage all the clinicians to be aware of the quality of life of patients and the development of long-term toxicities like second primary malignancies during the follow-up.

## Perspectives

Targeted therapies are increasingly used in HCL. In the future, these treatments or other new targeted therapies could be combined with each other or with conventional PNA. They could be developed in SBLPN, which are often refractory or have short-lasting responses to chemotherapy. Several clinical trials are ongoing (**Table 3**).

**Table 3:** Current clinical trials in HCL and HCL-like disorders.

Drugs	Disease	Phase	NCT Number	Status
**Obinutuzumab + Vemurafenib**	Untreated HCL	2	NCT03410875	Active, not recruiting
**Binimetinib**	BRAF wild type R/R HCL and HCL-V	2	NCT04322383	Recruiting
**Binimetinib + Encorafenib**	BRAF mutated R/R HCL	2	NCT04324112	Recruiting
**Moxetumomab pasudotox + Rituximab**	R/R HCL and HCL-V	1	NCT03805932	Recruiting
**Anti-CD22 CAR T-cells**	R/R HCL and HCL-V	1	NCT04815356	Recruiting

CAR T-cells, Chimeric antigen receptor T-cells, source: Clinicaltrials.gov.

Bcl2i are currently used in acute myeloid leukemia (AML) (93) and B-CLPD, especially in CLL (94–97). They are BH3 mimetics and induce apoptosis in leukemic cells. Preclinical data showed apoptosis of HCL cells exposed to venetoclax in *in vitro* experiments. However, in this study, signals from microenvironment protected HCL cells from venetoclax-induced apoptosis (13). Clinically, to our knowledge, only one case report described the use of venetoclax in HCL, and it was a highly atypical case (98).

Receptor tyrosine kinase-like orphan receptor 1 (ROR1) plays a major role in the embryonic development. It is also highly expressed at the surface of several cancer cells, especially in breast cancer, chronic myeloid leukemia, CLL, MCL, and DLBCL. It has been demonstrated that ROR1 is highly expressed on HCL cells as well (99). Thus, ROR1 could constitute a novel therapeutic target with the development of monoclonal antibodies, antibody drug conjugates, CAR T-cells or small molecule inhibitors (100). A part of the HCL2025 project, created by the *HCL Foundation* and the *Leukemia and Lymphoma Society*, is dedicated to the study of ROR1 as a novel therapeutic target in HCL.

Finally, other drugs are also in development such as new MEKi (Zapnometinib, Selumetinib, Mirdematinib, Refanitinib, Pimarsertib), inhibitors of cell cycle (Flavoperidol, Palbocilib, Abcmacilib), and epigenetic modifiers (5-azacitidine, Decitabine, Romidepsine, Belinostat, Panobinostat, EZH2 selective inhibitors: Tazemetostat or others).

## Author contributions

EM and XT designed the article, EM contributed essential bioinformatic tools, XT and JP wrote the paper, EM, JP and XT approved the manuscript. All authors contributed to the article and approved the submitted version.
